# Non-centrosymmetric Rb_2_Mn_2_(MoO_4_)_3_

**DOI:** 10.1107/S1600536814013099

**Published:** 2014-06-11

**Authors:** Chahira Bouzidi, Mohamed Faouzi Zid, Ahmed Driss, Amira Souilem

**Affiliations:** a Laboratoire de Matériaux et Cristallochimie, Faculté des Sciences de Tunis, Université de Tunis ElManar, 2092 Manar II Tunis, Tunisia

## Abstract

The title compound, dirubidium dimanganese(II) tris­(tetra­oxo­molyb­date), Rb_2_Mn_2_(MoO_4_)_3_, was prepared by solid-state reactions. The structure can be described as being composed of MnO_6_ octa­hedra sharing corners with MoO_4_ tetra­hedra. The three-dimensional framework contains cavities in which the rubidium ions are located. The Rb^+^ cations are within distorted nine- and 12-vertex polyhedra. The pairs of different Mn^2+^ and Rb^+^ cations are each located on threefold rotation axes.. Rb_2_Mn_2_(MoO_4_)_3_ is isotypic with compounds of the Cs_2_ *M*_2_Mo_3_O_12_ (*M* = Ni, Fe) family. A comparative structural description is provided between the structure of the title compound and those of related phases. Differences with structures such as alluaudite are discussed.

## Related literature   

For isotypic structures, see: Zolotova *et al.* (2011 ▶); Namsara­eva *et al.* (2011 ▶). For background to the physico-chemical properties of related compounds, see: Chaalia *et al.* (2012 ▶); Prabaharan *et al.* (1997 ▶); Ouerfelli *et al.* (2007 ▶); Hansen *et al.* (1988 ▶); Masquelier *et al.* (1995 ▶). For details of of the preparation and for structurally related compounds, see: Solodovnikov *et al.* (1986 ▶, 1998 ▶); Solodovnikov & Solodovnikova (1997 ▶); Tsyrenova *et al.* (2004 ▶); Bouzidi *et al.* (2014 ▶). For details of structurally different compounds, see: Brahim & Amor (2003 ▶); Warner *et al.* (1993 ▶); Korzenski *et al.* (1998 ▶); Chouaibi *et al.* (2001 ▶); Pertlik (1987 ▶); Antenucci *et al.* (1995 ▶); Zid *et al.* (2005 ▶); Hatert *et al.* (2004 ▶); Lii & Shih (1994 ▶). For bond lengths, see: Souilem *et al.* (2014 ▶); Frigui *et al.* (2012 ▶); Leclaire & Raveau (1988 ▶); Sebastian *et al.* (2003 ▶) and for bond-valence sums, see: Brown & Altermatt (1985 ▶).

## Experimental   

### 

#### Crystal data  

Rb_2_Mn_2_(MoO_4_)_3_
*M* *_r_* = 760.64
Cubic, 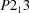

*a* = 10.9002 (9) Å
*V* = 1295.10 (19) Å^3^
*Z* = 4
Mo *K*α radiation
μ = 12.24 mm^−1^
*T* = 298 K
0.25 × 0.15 × 0.10 mm

#### Data collection  

Enraf–Nonius CAD-4 diffractometer
Absorption correction: ψ scan (North *et al.*, 1968 ▶) *T*_min_ = 0.071, *T*_max_ = 0.210
2949 measured reflections
916 independent reflections
838 reflections with *I* > 2σ(*I*)
*R*_int_ = 0.044
2 standard reflections every 120 min intensity decay: 1.3%

#### Refinement  

*R*[*F* ^2^ > 2σ(*F* ^2^)] = 0.035
*wR*(*F* ^2^) = 0.079
*S* = 1.10
916 reflections
59 parameters
Δρ_max_ = 1.03 e Å^−3^
Δρ_min_ = −0.96 e Å^−3^
Absolute structure: Flack (1983 ▶), 264 Friedel pairs
Absolute structure parameter: 0.04 (2)

### 

Data collection: *CAD-4 EXPRESS* (Duisenberg, 1992 ▶; Macíček & Yordanov, 1992 ▶); cell refinement: *CAD-4 EXPRESS*; data reduction: *XCAD4* (Harms & Wocadlo, 1995 ▶); program(s) used to solve structure: *SHELXS97* (Sheldrick, 2008 ▶); program(s) used to refine structure: *SHELXL97* (Sheldrick, 2008 ▶); molecular graphics: *DIAMOND* (Brandenburg & Putz, 2001 ▶); software used to prepare material for publication: *WinGX* (Farrugia, 2012 ▶).

## Supplementary Material

Crystal structure: contains datablock(s) I. DOI: 10.1107/S1600536814013099/ru2059sup1.cif

Structure factors: contains datablock(s) I. DOI: 10.1107/S1600536814013099/ru2059Isup2.hkl

CCDC reference: 1006923

Additional supporting information:  crystallographic information; 3D view; checkCIF report

## Figures and Tables

**Fig. 1.**
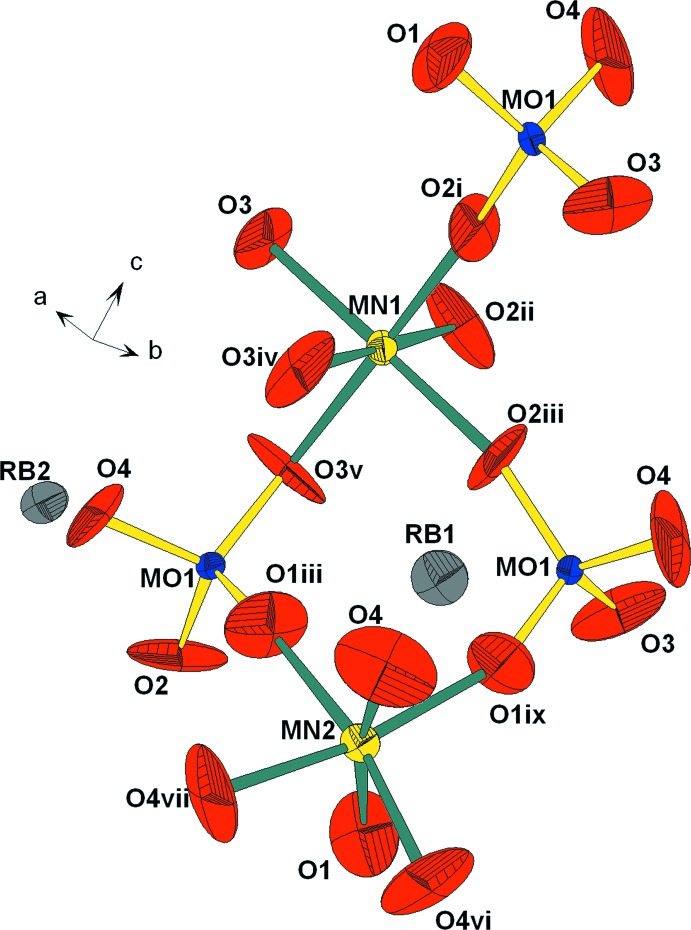
Unité asymétrique dans le composé Rb2Mn2Mo3O12. Les éllipsoïdes ont été définis avec 50% de probabilité. [Code de symétrie]: (i) y, z, 1 + x; (ii) 1/2 - x, 1 - y, z + 1/2; (iii) 1 - z, 1/2 + x, 3/2 - y; (iv) 1 + y, -1/2 + z, 3/2 - x; (v) 3/2 - z, 1 - x, 1/2 + y; (vi) 1 - x, 1/2 + y, 3/2 - z; (vii) y, z, x; (viii) z - 1/2, 3/2 - x, 1 - y; (ix) y - 1/2, 3/2 - z, 1 - x.

**Fig. 2.**
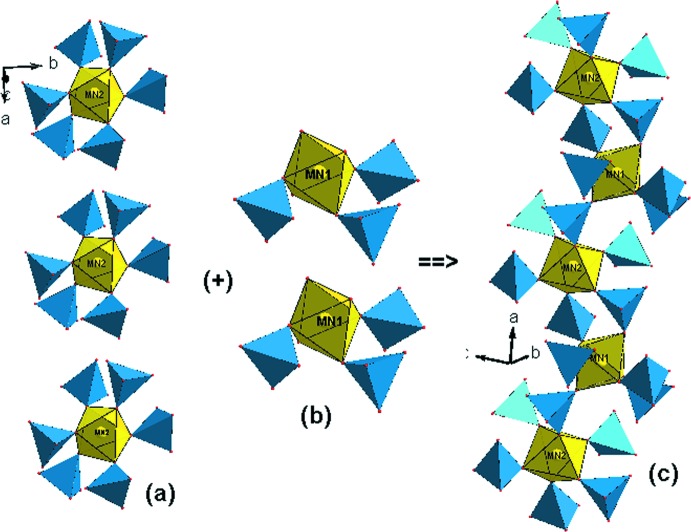
: (a) Projection de l'unité Mn(2)Mo6O24; (b) Projection de l'unité Mn(1)Mo3O12; (c) Projection d'une chaîne.

**Fig. 3.**
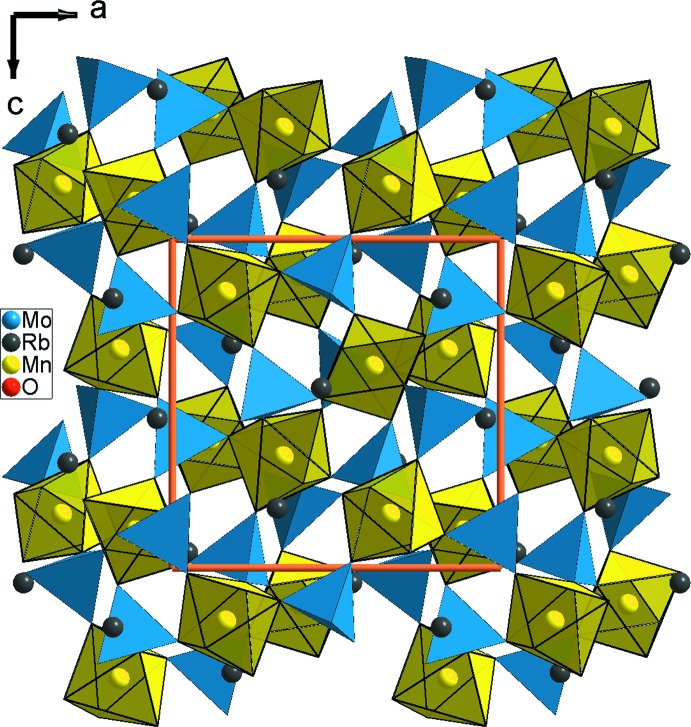
Projection de la structure de Rb2Mn2Mo3O12 selon b.

**Fig. 4.**
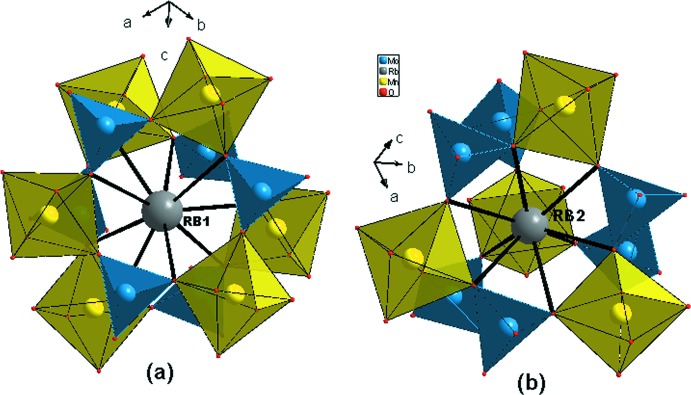
: (a) Représentation de l'environnement de Rb(1), (b) Représentation de l'environnement de Rb(2).

**Fig. 5.**
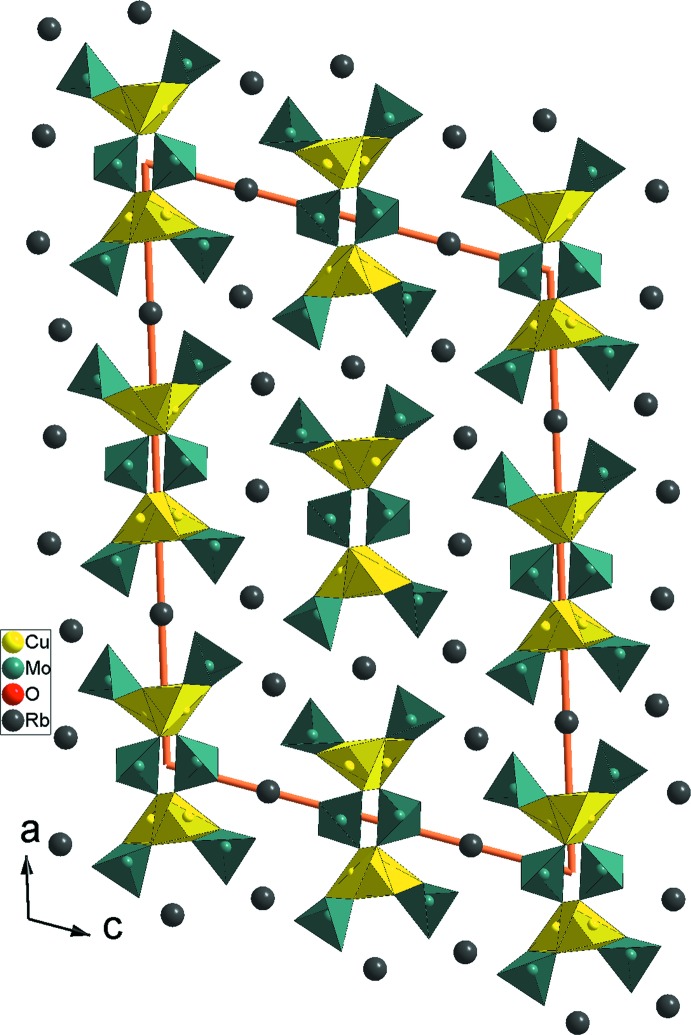
: Projection de la structure de Rb2Cu2Mo3O12 selon b.

**Fig. 6.**
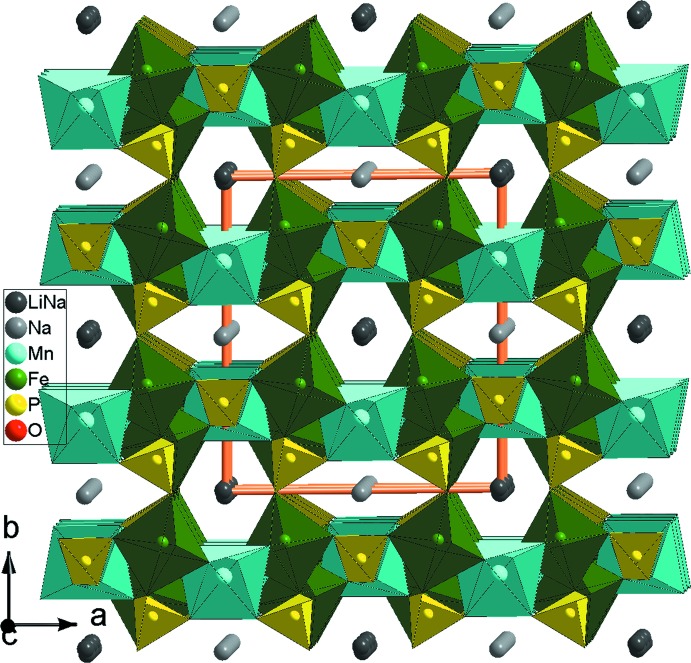
: Projection de la structure de Na0.5Li0.5MnFe2P3O12 type alluaudite, selon c.

## supplementary crystallographic information

### S1. Comment

Plusieurs travaux ont été consacrés à la recherche des matériaux inorganiques à charpentes ouvertes formées de tétraèdres et d'octaèdres. En effet, ils ont attiré l'attention de maints chercheurs grâce à leurs propriétés physico-chimiques notamment: conduction ionique (Chaalia *et al.*, 2012; Prabaharan *et al.*, 1997) et magnétique (Ouerfelli *et al.*, 2007; Masquelier *et al.*, 1995).

En particulier, les composés non centrosymétriques possédant des propriétés intéressantes plus précisément les propriétés d'optiques non-linéaires (ONL). En effet, ils permettent donc d'envisager différentes applications notamment le doublage de fréquences. Parmi les composés inorganiques cités dans la littérature l'orthophosphate KTiOPO_4_ (Hansen *et al.*, 1988). Dans le but d'accéder à de nouveaux matériaux de ce type, nous avons entrepris l'exploration des systèmes A–Mn–Mo–O (A = ion monovalent) dans lesquels différentes phases ont été précédemment isolées: K_2_Mn_2_Mo_3_O_12_ (Solodovnikov *et al.*, 1986), K_10_MnMo_7_O_27_, (Solodovnikov *et al.*, 1997), K_4_MnMo_4_O_15_ (Solodovnikov *et al.*, 1998), Ag_2_Mn_2_Mo_3_O_12_ (Tsyrenova *et al.*, 2004), Mn_2.17_Mo_3_Na_1.67_O_12_ (Bouzidi *et al.*, 2014). Une nouvelle phase non centrosymétrique de formulation Rb_2_Mn_2_(MoO_4_)_3_, a été synthétisée par réaction à l'état solide.

L'unité *asym*étrique dans Rb_2_Mn_2_(MoO_4_)_3_, renferme deux octaèdres MnO_6_ et trois tétraèdres MoO_4_ liés par mise en commun de sommets. La compensation de charge est assurée par les cations Rb^+^ (Fig. 1). Dans cette unité, nous remarquons la présence du groupement cyclique Mn_2_Mo_2_O_16_.

La structure est caractérisée par une charpente tridimensionnelle, dans laquelle chaque octaèdre Mn(2)O_6_ partage ses six sommets avec respectivement six tétraèdres MoO_4_ différents formant l'unité Mn(2)Mo_6_O_24_ (Fig. 2a). Dans la charpente anionique, l'insertion des groupements Mn(1)Mo_3_O_12_ (Fig. 2 b) entre les unités Mn(2)Mo_6_O_24_ renforce d'avantage leur connexion conduisant à des chaînes disposées selon la direction *a* (Fig. 2c). La jonction entre ces dernières, assurée par formation de ponts mixtes de type Mn–O–Mo conduit à une charpente tridimensionnelle possédant des cavités où résident les cations Rb^+^ (Fig. 3). Il est à signaler, que les cations Rb(1) e t Rb(2) sont entourés respectivement par douze et neuf atomes d'oxygène (Fig. 4a) et (Fig. 4b).

Les distances moyennes d(Mo–O), d(Mn–O) et (Rb–O) sont égales respectivement à 1,726 (2) Å, 2,136 (2) Å e t 3,274 (2) Å, conforment à celles observées dans la littérature (Souilem *et al.*, 2014; Sebastian *et al.*, 2003; Frigui *et al.*, 2012; Leclaire *et al.*, 1988).

De plus, l'utilisation de la méthode BVS, pour le calcul des différentes valences des liaisons, en utilisant la formule empirique de Brown (Brown & Altermatt, 1985) vérifie bien les valeurs attendues de charges des ions Mo1(6,14), Mn1(2,14), Mn2(2,11), Rb1(0,52), Rb2(0,74).

Un examen rigoureux de différentes structures trouvées dans la littérature révèle que le matériau étudié est isostructural à ceux de formulation Cs_2_*M*_2_Mo_3_O_12_ (*M*= Ni, Fe) (Zolotova *et al.*, 2011; Touyana *et al.*, 2011).

Les matériaux Cs_2_Co_2_Mo_3_O_12_ (Solodovnikov *et al.*, 1986) et Rb_2_Cu_2_Mo_3_O_12_ (Solodovnikov *et al.*, 1997) isomorphes à celle de notre composé présentent d'autres variétés cristallographiques. En effet, ils cristallisent respectivement dans le système orthorhombique groupe d'espace *P*2_1_2_1_2_1_ et dans le système monoclinique (GS: *C*2/*c*).

Une comparaison de la structure du composé étudié Rb_2_Mn_2_Mo_3_O_12_(a = 10.9002 (9) Å) avec celle de Rb_2_Cu_2_Mo_3_O_12_ (a= 27,698 Å, b= 5,102 Å, c= 19,292Å, β= 107,26 °)(Solodovnikov *et al.*, 1997) montre une différence nette dans la disposition des polyèdres dans la charpente anionique. En effet, on remarque que pour Rb_2_Cu_2_Mo_3_O_12_ les octaèdres Cu(1)O_6_ et Cu(2)O_6_ sont liés par mise en commun d'arêtes formant des chaînes d'octaèdres. Ces dernières sont connectées à leur tour par partage de sommets avec les tétraèdres Mo(1)O_4_ conduisant à des rubans disposées selon [010]. Il en résulte, dans le cas de Rb_2_Cu_2_Mo_3_O_12_, une structure unidimensionnelle (one-dimensional) où les cations Rb^+^ résident dans les espaces inter-rubans (Fig. 5).

La recherche de structures présentant des aspects communs avec celle de Rb_2_Mn_2_Mo_3_O_12_, nous a conduit à la famille des alluaudites de formule générale AA'*M*'*M*_2_(XO_4_)_3_ dont A et A' sont des alcalins et M et M' sont des métaux de transition (Brahim & Amor, 2003; Warner *et al.*, 1993; Korzenski *et al.*, 1998; Chouaibi *et al.*, 2001; Pertlik, 1987; Antenucci *et al.*, 1995; Zid *et al.*, 2005; Chaalia *et al.*, 2012; Hatert *et al.*, 2004; Lii & Shih, 1994) (Fig. 6). La comparaison a montré une différence significative dans la charpente et en particulier dans les modes de connection des polyèdres MO_6_. En effet, dans les structures alluaudites les octaèdres sont liés par partage d'arêtes formant des chaînes qui se lient aux tétraèdres XO_4_ par mise en commun de sommets pour conduire à des couches. Ces dernières se regroupent moyennant des ponts mixtes M–O–*X* pour donner des charpentes tridimensionnelles possédant des tunnels où logent les cations monovalents. On peut donc conclure que notre matériau diffère d'une forme alluaudite (Fig. 6).

### S2. Experimental

La synthèse a été faite par voie sèche à partir d'un mélange C_9_H_9_MnO_6_·2H_2_O (Fluka, 63538), (NH_4_)_2_Mo_4_O_13_ (Fluka, 69858) e t Rb_2_CO_3_, pris dans les proportions molaires Rb:Mn:Mo = 2:2:3. Le traitement thermique a été réalisé en deux étapes: la première consiste à préchauffer les réactifs à 673 K pendant 24 heures en vue d'éliminer les composés volatils. Puis, le résidu a été porté à 923 K (proche de la température de fusion) et il est porté à cette température pendant une semaine pour favoriser la germination et la croissance des cristaux. Le résidu final a subi en premier lieu un refroidissement lent (5 K/24 h) jusqu'à 900 K suivi d'un autre plus rapide (50 K/jour) jusqu'à la température ambiante. Des cristaux de couleur jaunâtre ont été séparés par l'eau chaude.

### S3. Refinement

*L*'affinement de tous les paramètres variables conduit à des ellipsoïdes bien définis. Les densités d'électrons maximum et minimum restants dans la Fourier-différence sont situées respectivement à 0,97 Å de O4 et à 0,39 Å de Rb2.

### Crystal data

**Table:**

Rb_2_Mn_2_(MoO_4_)_3_	*D*_x_ = 3.901 Mg m^−^^3^
*M**_r_* = 760.64	Mo *K*α radiation, λ = 0.71073 Å
Cubic, *P*2_1_3	Cell parameters from 25 reflections
Hall symbol: P 2ac 2ab 3	θ = 11–15°
*a* = 10.9002 (9) Å	µ = 12.24 mm^−^^1^
*V* = 1295.10 (19) Å^3^	*T* = 298 K
*Z* = 4	Prism, yellow
*F*(000) = 1384	0.25 × 0.15 × 0.10 mm

### Data collection

**Table:**

Enraf–Nonius CAD-4 diffractometer	838 reflections with *I* > 2σ(*I*)
Radiation source: fine-focus sealed tube	*R*_int_ = 0.044
Graphite monochromator	θ_max_ = 26.9°, θ_min_ = 2.6°
ω/2θ scans	*h* = −13→1
Absorption correction: ψ scan (North *et al.*, 1968)	*k* = −1→13
*T*_min_ = 0.071, *T*_max_ = 0.210	*l* = −13→6
2949 measured reflections	2 standard reflections every 120 min
916 independent reflections	intensity decay: 1.3%

### Refinement

**Table:**

Refinement on *F*^2^	Secondary atom site location: difference Fourier map
Least-squares matrix: full	*w* = 1/[σ^2^(*F*_o_^2^) + (0.0187*P*)^2^ + 14.7659*P*] where *P* = (*F*_o_^2^ + 2*F*_c_^2^)/3
*R*[*F*^2^ > 2σ(*F*^2^)] = 0.035	(Δ/σ)_max_ < 0.001
*wR*(*F*^2^) = 0.079	Δρ_max_ = 1.03 e Å^−^^3^
*S* = 1.10	Δρ_min_ = −0.96 e Å^−^^3^
916 reflections	Extinction correction: *SHELXL97* (Sheldrick, 2008), Fc^*^=kFc[1+0.001xFc^2^λ^3^/sin(2θ)]^-1/4^
59 parameters	Extinction coefficient: 0.0049 (4)
0 restraints	Absolute structure: Flack (1983), 264 Friedel pairs
Primary atom site location: structure-invariant direct methods	Absolute structure parameter: 0.04 (2)

### Special details

**Table:**

Geometry. All e.s.d.'s (except the e.s.d. in the dihedral angle between two l.s. planes) are estimated using the full covariance matrix. The cell e.s.d.'s are taken into account individually in the estimation of e.s.d.'s in distances, angles and torsion angles; correlations between e.s.d.'s in cell parameters are only used when they are defined by crystal symmetry. An approximate (isotropic) treatment of cell e.s.d.'s is used for estimating e.s.d.'s involving l.s. planes.
Refinement. Refinement of *F*^2^ against ALL reflections. The weighted *R*-factor *wR* and goodness of fit *S* are based on *F*^2^, conventional *R*-factors *R* are based on *F*, with *F* set to zero for negative *F*^2^. The threshold expression of *F*^2^ > σ(*F*^2^) is used only for calculating *R*-factors(gt) *etc*. and is not relevant to the choice of reflections for refinement. *R*-factors based on *F*^2^ are statistically about twice as large as those based on *F*, and *R*- factors based on ALL data will be even larger.

### Fractional atomic coordinates and isotropic or equivalent isotropic displacement parameters (Å^2^)

**Table:**

	*x*	*y*	*z*	*U*_iso_*/*U*_eq_	
Mo1	0.37706 (7)	0.52435 (7)	0.79714 (7)	0.0157 (2)	
Mn1	0.38791 (13)	0.61209 (13)	1.11209 (13)	0.0197 (5)	
Mn2	0.33762 (13)	0.83762 (13)	0.66238 (13)	0.0227 (6)	
Rb1	0.04628 (12)	0.54628 (12)	0.95372 (12)	0.0487 (6)	
Rb2	0.31889 (10)	0.18111 (10)	0.81889 (10)	0.0371 (5)	
O1	0.3993 (13)	0.6707 (9)	0.7457 (11)	0.083 (4)	
O2	0.2720 (11)	0.4448 (11)	0.7079 (10)	0.083 (4)	
O3	0.3323 (11)	0.5164 (12)	0.9489 (8)	0.084 (4)	
O4	0.5171 (12)	0.4546 (14)	0.7765 (13)	0.109 (5)	

### Atomic displacement parameters (Å^2^)

**Table:**

	*U*^11^	*U*^22^	*U*^33^	*U*^12^	*U*^13^	*U*^23^
Mo1	0.0163 (4)	0.0156 (4)	0.0152 (4)	0.0025 (3)	−0.0033 (3)	−0.0012 (3)
Mn1	0.0197 (5)	0.0197 (5)	0.0197 (5)	0.0016 (6)	0.0016 (6)	−0.0016 (6)
Mn2	0.0227 (6)	0.0227 (6)	0.0227 (6)	−0.0053 (6)	0.0053 (6)	0.0053 (6)
Rb1	0.0487 (6)	0.0487 (6)	0.0487 (6)	−0.0082 (6)	0.0082 (6)	0.0082 (6)
Rb2	0.0371 (5)	0.0371 (5)	0.0371 (5)	0.0042 (5)	−0.0042 (5)	0.0042 (5)
O1	0.113 (10)	0.041 (5)	0.096 (9)	−0.022 (7)	−0.024 (8)	0.029 (6)
O2	0.099 (9)	0.098 (9)	0.051 (6)	−0.054 (7)	−0.058 (6)	0.026 (6)
O3	0.102 (9)	0.128 (10)	0.021 (4)	−0.055 (8)	0.022 (5)	−0.033 (6)
O4	0.084 (9)	0.129 (11)	0.113 (11)	0.089 (9)	−0.019 (8)	0.004 (9)

### Geometric parameters (Å, º)

**Table:**

Mo1—O1	1.708 (10)	Rb1—O3^iii^	3.135 (12)
Mo1—O4	1.721 (11)	Rb1—O3^ix^	3.135 (12)
Mo1—O3	1.726 (8)	Rb1—O2^ii^	3.407 (13)
Mo1—O2	1.734 (10)	Rb1—O2^x^	3.407 (13)
Mn1—O2^i^	2.125 (10)	Rb1—O2^xi^	3.407 (13)
Mn1—O2^ii^	2.125 (10)	Rb1—O4^x^	3.586 (13)
Mn1—O2^iii^	2.125 (10)	Rb1—O4^ii^	3.586 (13)
Mn1—O3	2.150 (9)	Rb1—O4^xi^	3.586 (13)
Mn1—O3^iv^	2.150 (9)	Rb2—O1^xii^	3.153 (15)
Mn1—O3^v^	2.150 (9)	Rb2—O1^xiii^	3.153 (15)
Mn2—O4^vi^	2.139 (10)	Rb2—O1^iv^	3.153 (15)
Mn2—O4^vii^	2.139 (10)	Rb2—O2^xiv^	3.160 (12)
Mn2—O4^viii^	2.139 (10)	Rb2—O2	3.160 (12)
Mn2—O1^ix^	2.142 (10)	Rb2—O2^x^	3.160 (12)
Mn2—O1^iii^	2.142 (10)	Rb2—O4^xii^	3.221 (17)
Mn2—O1	2.142 (10)	Rb2—O4^iv^	3.221 (17)
Rb1—O3	3.135 (12)	Rb2—O4^xiii^	3.221 (17)
			
O1—Mo1—O4	104.1 (7)	O2^iii^—Mn1—O3^v^	91.9 (4)
O1—Mo1—O3	113.7 (6)	O3—Mn1—O3^v^	88.2 (5)
O4—Mo1—O3	110.7 (6)	O3^iv^—Mn1—O3^v^	88.2 (5)
O1—Mo1—O2	112.1 (5)	O4^vi^—Mn2—O4^vii^	88.6 (5)
O4—Mo1—O2	107.0 (7)	O4^vi^—Mn2—O4^viii^	88.6 (5)
O3—Mo1—O2	109.0 (6)	O4^vii^—Mn2—O4^viii^	88.6 (5)
O2^i^—Mn1—O2^ii^	88.8 (5)	O4^vi^—Mn2—O1^ix^	168.3 (6)
O2^i^—Mn1—O2^iii^	88.8 (5)	O4^vii^—Mn2—O1^ix^	98.2 (5)
O2^ii^—Mn1—O2^iii^	88.8 (5)	O4^viii^—Mn2—O1^ix^	82.1 (6)
O2^i^—Mn1—O3	179.2 (5)	O4^vi^—Mn2—O1^iii^	82.1 (6)
O2^ii^—Mn1—O3	91.9 (4)	O4^vii^—Mn2—O1^iii^	168.3 (6)
O2^iii^—Mn1—O3	91.0 (6)	O4^viii^—Mn2—O1^iii^	98.2 (5)
O2^i^—Mn1—O3^iv^	91.9 (4)	O1^ix^—Mn2—O1^iii^	92.3 (6)
O2^ii^—Mn1—O3^iv^	91.0 (6)	O4^vi^—Mn2—O1	98.2 (5)
O2^iii^—Mn1—O3^iv^	179.2 (5)	O4^vii^—Mn2—O1	82.1 (6)
O3—Mn1—O3^iv^	88.2 (5)	O4^viii^—Mn2—O1	168.3 (6)
O2^i^—Mn1—O3^v^	91.0 (6)	O1^ix^—Mn2—O1	92.3 (6)
O2^ii^—Mn1—O3^v^	179.2 (5)	O1^iii^—Mn2—O1	92.3 (6)

Symmetry codes: (i) *y*, *z*, *x*+1; (ii) −*x*+1/2, −*y*+1, *z*+1/2; (iii) −*z*+1, *x*+1/2, −*y*+3/2; (iv) −*y*+1, *z*−1/2, −*x*+3/2; (v) −*z*+3/2, −*x*+1, *y*+1/2; (vi) −*x*+1, *y*+1/2, −*z*+3/2; (vii) *y*, *z*, *x*; (viii) *z*−1/2, −*x*+3/2, −*y*+1; (ix) *y*−1/2, −*z*+3/2, −*x*+1; (x) −*y*+1/2, −*z*+1, *x*+1/2; (xi) −*z*+1/2, −*x*+1, *y*+1/2; (xii) −*x*+1, *y*−1/2, −*z*+3/2; (xiii) −*z*+1, *x*−1/2, −*y*+3/2; (xiv) *z*−1/2, −*x*+1/2, −*y*+1.
